# Going Abroad and Going Green: The Effects of Top Management Teams’ Overseas Experience on Green Innovation in the Digital Era

**DOI:** 10.3390/ijerph192214705

**Published:** 2022-11-09

**Authors:** Shuang Meng, Pengxiang Wang, Jiajie Yu

**Affiliations:** 1 School of International Trade and Economics, Central University of Finance and Economics, Beijing 100081, China; 2 Business School, Beijing Normal University, Beijing 100875, China

**Keywords:** green innovation, top management team, overseas experience, digital economy

## Abstract

Green innovation has become one of the most important approaches to achieving sustainable development in modern business. Top management team (TMT)’s overseas experience, as one type of unique resources, constitutes the cognitive basis of the team and thus influences firms’ strategic decision-making. Based on the upper echelon theory, this study aims to investigate the effect of TMT’s overseas experiences on green innovation performances. By utilizing a panel dataset of Chinese listed firms, this study shows that TMTs’ overseas experience indeed promotes firms’ green innovation performance and that both firms’ digital transformation and regions’ digital economy development positively moderate the relationship between TMTs’ overseas experience and green innovation. These findings not only help managers better organize the TMT and green innovation strategy but also draw policymakers’ attention to the importance of the digital economy and sustainable development.

## 1. Introduction

With rapid economic development in the past decades, the excessive use of nonrenewable resources has damaged the atmosphere and exacerbated many environmental problems [1]. To save energy and reduce carbon emissions, the United Nations has created sustainable development goals (SDGs), and many countries have declared their commitment to environmentally friendly and sustainable activities [2]. The pressure of resource shortages and environmental pollution makes it difficult to maintain the growth mode of the traditional manufacturing sectors. As the demand for environmentally friendly technologies has gradually increased, major polluters such as manufacturing firms have become the focus of public attention. Firms in developing countries have become urgently required to change their business models to achieve green and sustainable development. Sustainable development has been discovered to be fundamentally dependent on upgrading and innovations [2,3]. Consequently, green innovation has become a significant component of firms’ promotion of green transformation [4,5]. Green innovation refers to the measures taken by firms to bring technological practices to bear on environmental pollution and actively reduce environmental problems caused by their production and operation activities [6,7]. However, green innovation has unique dual externalities (i.e., knowledge spillover and environmental protection externalities), combined with the inherent characteristics of traditional innovation activities, such as high risks and a long and unpredictable investment return cycle [8,9], which means that it is treated as a balance between environmental sustainability and economic interests that determines the difficulty of implementation. According to the Porter hypothesis, stringent environmental regulations can induce efficiency and facilitate innovations that can improve competitive advantages. Therefore, green innovation has become a significant strategic concern for top management teams (TMTs) in practice and has attracted research attention in recent years [5,10]. Particularly, researchers have studied how TMT characteristics affect firms’ green innovation performance. We seek to extend this stream of literature in the context of China.

Over the past decade, another significant change in economic development has been the expansion of a wide range of digital technologies and digital infrastructures, which have reshaped all aspects of business and organizations [11,12]. In addition, the development of the digital economy (i.e., digital platforms, digital technologies, and digital infrastructure) has been crucial for sustainable growth in modern society [13,14,15]. These phenomena have attracted the attention of researchers and practitioners [14,16,17,18]. However, how firms’ digital transformation efforts and regions’ digital economy development affect green innovation and how they are involved in the strategic processes of TMTs have not yet been investigated in previous studies.

This study aims to answer the following questions: What are the effects of TMTs’ overseas experience on firms’ green innovation performance? How do firms’ digital transformation and regions’ digital economy development moderate the relationship between TMTs’ overseas experience and green innovation in the digital era? This study employs a panel dataset of Chinese A-share listed firms from 2011 to 2018 to conduct the empirical analysis. It is found that TMT’s overseas experience exerts positive impacts on green innovation and that the positive relationship between TMT’s overseas experience and green innovation can be positively moderated by digital transformation and digital economy development. In addition to micro-level data on firm characteristics, we further incorporate macro-level data on regional digital economy development into this study. The combination of both micro- and macro-level data in the empirical analysis allows us to scrutinize the moderating effects of firms’ digital transformation and regional digital economy development, which have not been simultaneously and systematically investigated in previous studies. Our findings provide a theoretical basis and practical implications for firms to adjust the structure and strategic choices of TMTs effectively to drive green innovation development.

The contributions of this study to the existing literature are threefold. First, we extend the literature on the antecedents and determinants of firms’ green innovation performance. In particular, we contribute to the determinants of green innovation at the firm level, that is, the specific characteristics of TMT and the context of the digital era. Second, this study adds to the previous literature on TMTs and their strategic outcomes. In particular, we contribute to the research stream on upper echelons theory by providing empirical evidence for the relationship between the TMT’s overseas experience and firms’ green innovation performance. Finally, we highlight the importance of digital transformation and digital economy development in the process of TMT decision-making, which has not been systematically studied in previous studies. Although some of the recent studies have explored the relationship between the characteristics of TMT and firm performance in the digital age [19,20], this study differs from their work in two major aspects. On the one hand, by utilizing a panel dataset of Chinese listed firms, we focus on firms’ sustainable development from the perspective of green innovation. On the other hand, we specifically examine the moderating effects of firms’ digital transformation and regional digital economy development, which contribute to existing studies by shedding both micro- and macro-level empirical light on this topic.

The remainder of the study is organized as follows. Section 2 provides the literature review and develops three hypotheses. Section 3 presents the data and methodology used. Section 4 presents the empirical results and robustness checks. Section 5 discusses the findings and provides theoretical and practical implications. Finally, Section 6 concludes the study.

## 2. Literature Review and Hypothesis Development

### 2.1. Green Innovation and Its Determinants

This study is related to the literature on the strategic roles and determinants of green innovation. Green innovation has been defined as the measures taken by firms to bring technological practices to bear on environmental pollution and reduce environmental problems caused by their production and operation activities [6,7], which is often characterized by important environmental dimensions, such as environmental protection, energy minimization, materials reduction, and pollution prevention [4,21]. Green innovation holds considerable economic significance and can effectively promote the improvement of brand equity [22], help firms establish an environmentally friendly image and expand market share [5,23] so that firms can better cope with the challenges of competitors and achieve better financial performance [24,25]. As a kind of environmentally friendly innovation, green innovation can effectively meet the needs of high-quality economic development. Therefore, green innovation has gradually become a development force for firms in developing countries to gain competitive advantages that cannot be ignored [26,27]. However, innovation includes not only technological activities but also organizational improvements [3], which are characterized by long investment cycles and high risks. Compared with general innovation, green innovation requires a larger stock of capital and internal and external knowledge, and higher technological advantages [6,25], which means that such innovation is often treated as a balance between environmental sustainability and economic interests.

There are many previous studies that have examined the driving forces behind firms’ green innovation and eco-innovation [28,29,30,31,32,33]. In particular, several studies have investigated the determinants in the context of developing countries, which can provide valuable insights for sustainable development in emerging economies [26,27,34,35]. In principle, the driving factors of green innovation can be divided into the following categories. The first is the market factor, including market pressure and consumers’ green demand [36,37]. The second is that policies and regulations can effectively promote green technology innovation, and government subsidies and preferential tax policies can promote green innovation in the growth and transformation stages [9,38]. In addition, the recent literature has documented the relationship between sustainable business performance and enterprise digital transformation from the perspectives of knowledge co-creation [39], integrated sustainable urban technologies [40], and environmentally responsible behavior [41]. In addition to these factors, many studies have focused on firm-level characteristics, including corporate governance, strategy, and TMTs [36,37,42]. In summary, both internal knowledge and the external environment can influence green innovation activities. In this study, we focus on one significant aspect of the TMT, overseas experience, to discover how it affects firms’ green innovation performance.

### 2.2. TMTs’ Overseas Experience and Green Innovation

This study is also related to the literature on how TMT affects firms’ strategic decisions and performance. It has been found that TMT participates in the firm’s overall strategic decisions and formulates its operational direction, which is an essential part of firm innovation and development [43,44,45]. Recently, literature based on upper echelon theory has explored how TMTs’ characteristics affect firms’ green innovation performance. The upper echelon theory is rooted in Hambrick and Mason [46], which state that the growth environment, professional experience, educational background, and functional background of TMT members significantly affect firms’ strategic decision-making processes and performance [46,47,48,49,50]. Research in this field has focused mainly on the demographics of TMTs as a proxy for their knowledge level. Among these factors, the TMT’s overseas background is generally considered to be an important feature affecting firms’ innovation behavior and has attracted the attention of many scholars. Based on previous studies [51,52], TMT’s overseas experience is defined as prior educational or working experience for more than one year outside the home country.

Upper echelon theory provides a good angle to investigate the relationship between TMT’s overseas experience and green innovation. Upper echelon theory reveals that psychological and other observable characteristics of TMT members shape their values and knowledge and further influence their behaviors, such as identifying opportunities and risks, processing information, and formulating strategies, which results in shaping the strategic decisions and affecting firm performance [10,53,54,55]. TMTs with overseas experience often have better abilities and knowledge in international markets, global networks, creativity, problem-solving, and information processing [48,54]. Drawing on the insights from the upper echelon theory, these competencies and abilities can be a source of knowledge and competitive advantages because they are valuable, rare, and inimitable [10,48,50,52]. Thus, TMT overseas experience will help TMTs make informed decisions and control the risks that are associated with the decisions [56,57]. Next, considering the characteristics of green innovation, we further analyze why TMTs’ overseas experience may affect firms’ green innovation performance.

Firstly, the challenging and complex tasks of green innovation require the orientation and decisions of executives with higher risk tolerance and confidence. TMT members with overseas backgrounds often master cutting-edge science and technology and advanced management experience, which has a high spillover effect on firms and can effectively enhance their risk tolerance and technological innovation ability [45,51]. Compared with local executives, TMTs with overseas experience have more advantages regarding innovation and risk preference in decision-making [47,58]. Therefore, they tend to have more confidence and incentives than local executives, and can drive their firm to upgrade green innovation [56].

Secondly, since green innovation is treated as a balance between economic interests and environmental sustainability, strategic decision-making necessitates moral courage from decision-makers. Previous literature has documented the impacts of corporate social responsibility and environmental protection on green innovation in both developed [59] and developing countries [26,27]. Compared with local executives, TMT members with overseas experience may regard environmental and social responsibility issues as fundamental to the firm; thus, they may prefer to make the firm engage in green innovation activities. When participating in the decision-making process, TMT members with overseas experience tend to pay more attention to advanced innovation techniques to obtain investment returns and emphasize firm sustainability [52]. Thus, TMT members with overseas experience are likely to make the firm engage in green innovation.

Taken together, we argue that the TMT’s overseas experience is crucial in shaping green innovation performance. Therefore, the first hypothesis is proposed:

**H1.**  *TMTs’ overseas experience positively affects firms’ green innovation performance*.

### 2.3. The Moderating Roles of Digital Economy

In recent decades, digital economy has developed rapidly worldwide and it has attracted the attention of both scholars and practitioners [11]. Currently, digital technology, primarily carried by the internet, is applied in fields such as big data technology, blockchain technology, artificial intelligence technology, cloud computing technology, and digital technology applications, which have penetrated business and innovation activities. Many firms have developed digital technologies to embed value creation and realization into their production, R&D, and business processes [8]. Governments in every country pay special attention to the crucial role of the digital economy in economic development. Goldfarb and Tucker [11] is one of the most significant perspective paper to analyze whether and how digital technology affects economic and business activities. It has been discussed that the development of the digital economy has penetrated most aspects of business activities, affecting not only the strategic outcome but also the TMT’s decision-making process.

This study is also related to the literature on digital economy and innovation performance. First, digital technology can promote the connection between the upstream and downstream innovation partners and expand the innovation network boundaries in an innovation chain [11]. Second, digital technology can efficiently identify innovative opportunities and unique resources and reduce the cost of firm opportunities and resource searches [11,15,18,60]. Finally, digital economy development can help firms efficiently connect with domestic and foreign capital and product markets, accurately capture market trends, accelerate the transformation of firms’ production, and realize value-added benefits, thus providing power for sustainable innovation [13,17,61]. Moreover, several recent studies have investigated green product innovation and environmental innovation strategies in the context of the digital economy, focusing on data-driven Internet of Things systems [62], computationally networked urbanism [63], and employee green behavior [64]. Such studies offer valuable knowledge by providing TMT with new insights into improving firms’ environmental sustainability and green innovation performance.

Next, we discuss how the digital economy, in terms of firm-level digital transformation and region-level digital economy development, affects the impacts of the overseas experience on firms’ green innovation.

#### 2.3.1. The Moderating Role of Firm Digital Transformation

We make arguments regarding firm-level digital economy development in terms of digital transformation, which refers to the integration of digital technologies into business processes [18,65]. Firms’ digital transformation can improve information communication efficiency and strengthen internal and external production networks in the innovation process [13,18,60]. On the one hand, digital facilities and training, brought by digital transformation, can accelerate information flow within firms, improve information transparency, and enable firms to effectively participate in innovation cooperation across firm boundaries [17]. On the other hand, digital transformation can promote cooperation within innovation networks, thus reducing information asymmetry caused by geographical distance or cultural distance.

Compared to working in firms with lower-level digital transformation, TMT members in firms with higher-level digital transformation are more likely to drive the green innovation strategy for the following reasons. Firstly, a higher level of digital transformation in the firm would pave the way for the TMT to conduct such high-risk strategic decisions because of its higher efficiency and faster access to innovation resources as discussed above. Secondly, since TMTs with overseas experience may have limited connections in local networks [52], TMTs with overseas experience may find it easier to promote green innovation with the advantages of digital transformation in terms of easier and faster access to innovation networks.

Taken together, the second hypothesis is proposed:

**H2.**  *Digital transformation of the firm positively moderates the relationship between TMTs’ overseas experience and green innovation performance*.

#### 2.3.2. The Contingencies of Regional Digital Economy Development

Region-level digital economy development can be discussed in terms of Internet development, digital infrastructure development, and digital finance [66]. Firstly, region-level internet development can help TMT members examine the external environment, identify potential opportunities and risks, process information, and make strategies easier and faster. Secondly, region-level digital infrastructure can reduce communication costs among partners in the same or neighboring regions. Finally, digital finance development in the region has been discovered to affect firms’ green innovation because of its cost-reducing and efficiency-improving mechanisms [16,67].

Compared with working in firms located in low-level digital development regions, TMT members may benefit more from higher-level digital development when conducting green innovation strategies for the following reasons. Firstly, because TMTs with overseas experience have more advantages in top-tier knowledge, they are able to lead the change in the digital era [20]. Therefore, they are able to benefit more from the development of regional-level digital economy and better promote green innovation performance. Secondly, because TMTs with overseas experience may have disadvantages in local networking and local experience, the development of digital economy may mitigate these disadvantages by reducing costs and information asymmetries.

Thus, we propose the third hypothesis:

**H3.**  *Region-level digital economy development positively moderates the relationship between TMTs’ overseas experience and green innovation performance*.

**Figure 1:**
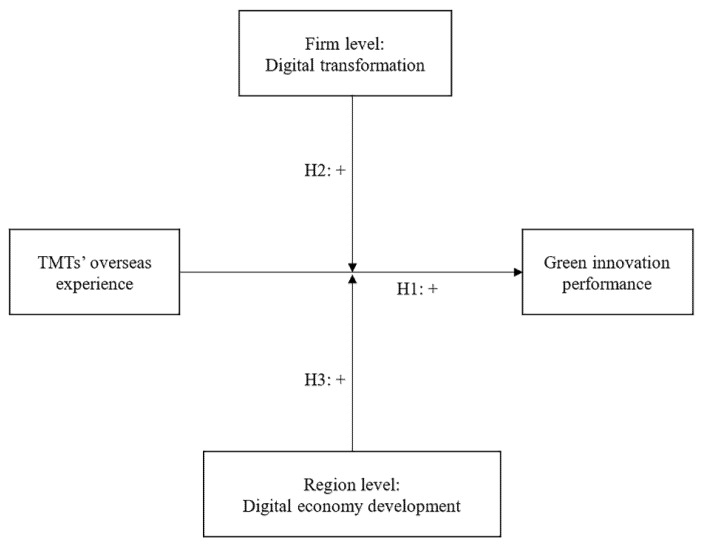
Conceptual framework.

To sum up, the logic between research hypotheses in this study is as follows. On one hand, according to the upper echelon theory, firms’ strategic decisions and innovation activities depend largely on the discretion of TMTs. Since overseas experience is playing an important role in shaping TMTs’ values and knowledge, which can promote firms’ green innovation performance. Hence, the first hypothesis indicates that TMTs’ overseas experience positively affects firms’ green innovation performance. On the other hand, digital technology has been documented as a significant factor in improving communication efficiency and facilitating the formation of green innovation networks. As two key dimensions of digital economy, firms’ digital transformation and regions’ digital economy development, tend to strengthen the relationship between TMTs’ overseas experience and green innovation. Thus, the second and third hypotheses state that the relationship between TMTs’ overseas experience and green innovation performance will be positively moderated by firms’ digital transformation and regions’ digital economy development, respectively. With the hypotheses proposed, the conceptual framework is constructed in Figure 1. The data and methodology used are discussed in the following sections.

## 3. Data and Methodology

### 3.1. Sample and Data Sources

This study uses data on Chinese listed firms from 2011 to 2018, which are sourced from the WIND and China Stock Market Accounting Research (CSMAR) databases. These two databases provide financial information, innovation, digitalization, and background information on TMTs, which have been widely adopted in previous studies on Chinese firms [52,68]. In addition, we construct the city-level digital economy development index by using data from the China Urban Statistical Yearbook. Note that the city-level digital economy development index is computed annually from 2011 to 2018. This study also extracts some data, including annual report data, to check, correct, or delete incorrect data. To improve the reliability and validity, the initial samples are processed as follows. First, ST, *ST, and delisted companies are eliminated (ST denotes “special treatment”. If a firm have negative net incomes or have other abnormal financial status for over two consecutive years, it will be under special treatment by stock exchanges. *ST indicates that the firm have negative net incomes or have other abnormal financial status for three consecutive years and receive delisting warning). Second, variables are processed to winsorize extreme variables at 1% and 99% to reduce the estimation bias from outliers. Finally, the sample is a panel dataset with 16,246 firm-year observations.

### 3.2. Variables

#### 3.2.1. Dependent Variable

Green innovation (GI). Existing research has measured the level of green innovation in terms of energy consumption and new products, which focuses mainly on macro-level analysis and cannot accurately measure firm-level green innovation. Following previous research [13], the logarithm of the total number of firms’ green patent applications is adopted to measure firms’ green innovation performance in the year. Taking the logarithmic transformation allows us to reduce the effects of outliers and to deal with heteroscedasticity, which improves the validity of empirical analyses.

#### 3.2.2. Independent Variable

TMT overseas experience (Overseas). In this study, board members and senior management team members are regarded as TMT members. Following previous research [69], a continuous variable—the proportion of TMT members with overseas experience to the total number of TMT members- is used to measure TMT overseas experience.

#### 3.2.3. Moderators

Firm digitization (FD). Following previous studies [8,70], firm digitization is the natural logarithm of the total number of digital transformation-related words appearing in listed firms’ annual reports. This variable can be sourced from the CSMAR. The variable is operationalized through dictionary-based text analysis of the listed firms’ annual reports in the following steps: First, keywords associated with digital transformation, including blockchain technology, big data technology, artificial intelligence technology, and cloud computing technology are identified. Second, machine-learning methods are used to obtain the frequency counts of these keywords in the firms’ annual reports.

Regional digital economy development (RD). As there is no census on any single-dimension index to characterize regional digital economy development precisely, this study develops a composite index. Following previous literature [66], this study uses principal component analysis (PCA) to construct a composite index. The subindex includes the following: (1) Number of broadband Internet access users per hundred people. (2) Proportion of computer service and software industry employees. (3) Total telecom business per capita. (4) Number of mobile phone users per hundred people. (5) China Digital Financial Inclusion Index, calculated by the Peking University Digital Finance Research Center and Ant Financial Group. The data above are quantifiable with high dimension and each indicator may have high dependence. The central idea of performing PCA is to reduce the dimensionality of the data, increase interpretability, and minimize information loss. This is accomplished by linearly transforming the data into a new coordinate system where the variation in the data can be characterized with fewer dimensions than the initial data. The regional digital economy development index RD is obtained through PCA.

#### 3.2.4. Control Variables

We control for variables that may affect green innovation, which have also been controlled in previous studies [13,15,37,52,71]. Firm size is measured by the logarithm of total assets because larger firms may have more resources to conduct green innovation [37]. Firm age is measured by the logarithm of the number of years a firm has been established. ROA is measured by the return on total assets because the profitability level may affect TMTs’ decision to conduct green innovation. *R&D intensity* is measured by the share of annual R&D investment in annual operating revenue as innovation outcome is always related to R&D input. Foreign sales are measured by the ratio of overseas revenue to total revenue as firms’ internationalization may affect their green innovation performance [54]. CEO duality is a dummy variable that takes the value of 1 if the CEO and chairman are the same person and 0 otherwise [10]. Fund ownership is measured by the share of ownership of funds because fund ownership may affect firms’ innovation decisions as key stakeholders [13]. In addition, we also control for industry competition by using one minus the HHI index in that industry competition may affect firms’ innovation decisions and outcomes through various mechanisms. Descriptions of all variable definitions are presented in Table 1.

### 3.3. Empirical Model Specification

We have utilized the panel data models to analyze the two-dimensional dataset which consists of “firm-year” observations. Compared with cross-sectional data and time-series data, panel data provide more variability, more efficiency, and less collinearity among the variables. The econometric methods allow us to exploit the variations across firms and over time, which help us to obtain unbiased estimations [72,73,74].

To test our hypotheses, this study constructs the following empirical model:

(1) $$GI_{i,t}=\alpha_{0}+\alpha_{1}Overseas_{i,t-1}+\alpha_{2}FD_{i,t-1}+\alpha_{3}RD_{r,t-1}+\sum \gamma_{i}Controls_{i,t-1}+FE+\varepsilon_{i,t}$$

where $GI_{i,t}$ is the green innovation performance of firm i in year t. $Overseas_{i,t-1}$ is the independent variable TMTs’ overseas experience of firm i in year t−1. $FD_{i,t-1}$ is the moderating variable and represents digital transformation firm i in year t−1. $RD_{r,\;t-1}$ is another moderating variable that indicates the digital economy development in city *r* where the firm is located in year t−1. $Controls_{i,t-1}$ is a set of control variables. $\varepsilon_{i,t}$ is the random error term. In addition, industry and year-fixed effects are added to control for unobserved heterogeneity at the industry and city levels. Following previous studies in TMT and strategy [58,75], we lag all independent variables for one year to alleviate reverse causality concerns.

In addition, following the existing literature [76,77,78], to test the moderating effect of firm digital transformation and regional digital economy development, this study introduces the interaction terms $Overseas_{i,t-1}\times FD_{t-1}$ and $Overseas_{i,t-1}\times RD_{i,t-1}$ into Model (1):

(2) $$GI_{i,t}=\alpha_{0}+\alpha_{1}Overseas_{i,t-1}+\alpha_{2}FD_{i,t-1}+\alpha_{3}RD_{r,t-1}+\alpha_{4}Overseas_{i,t-1}\times FD_{t-1}+\sum \gamma_{i}Controls_{i,t-1}+FE+\varepsilon_{i,t}$$

(3) $$GI_{i,t}=\alpha_{0}+\alpha_{1}Overseas_{i,t-1}+\alpha_{2}FD_{i,t-1}+\alpha_{3}RD_{r,t-1}+\alpha_{4}Overseas_{i,t-1}\times RD_{t-1}+\sum \gamma_{i}Controls_{i,t-1}+FE+\varepsilon_{i,t}$$

## 4. Results

### 4.1. Descriptive Statistics

In Table 2, we present the descriptive statistics for each variable. As indicated in the tables, large variations in the dependent variables and independent variables make it possible to fully explore how TMTs’ overseas experience affects firms’ green innovation performance.

Next, the correlation matrix between the variables and their significance is shown in Table 3. The correlation among the independent variable, moderators and control variables are below 0.3. Additionally, we also calculate the variance inflation factor (VIF) for all variables and found that all VIF values are smaller than the threshold value of 10. This evidence suggests that multicollinearity is not a serious concern in our research.

### 4.2. Empirical Results

#### 4.2.1. Baseline Regression Results

As in Table 4, the baseline regression results are presented. Model 1 includes only the control variables. Model 2 adds the independent variable, and Model 3 adds the moderators. As seen from the regression results in Model 2 of Table 4, the coefficient of the independent variable *Overseas* is 0.128 and is significant at the 5% significance level. The results are consistent with Models 2 and 3, which supports H1.

Among the control variables, the coefficients of *Firm size* are all positive and pass the 1% significance level test, which indicates that with the increase in firm scale, the green innovation performance of the firm increases. The coefficients of *Firm age* are significantly negative at the 5% level, indicating that the older the firm, the lower its green innovation performance. The coefficients of *ROA* are significantly positive at 5% level, which indicates that a higher level of financial performance leads to better green innovation performance. The coefficients of *R&D intensity* of firms are significantly positive at the 1% level, indicating that higher *R&D intensity* improves the green innovation performance of the firm. The coefficients of *CEO duality* are significantly positive at 10% level, indicating that *CEO duality* contributes to green innovation. The coefficients of *Fund ownership* are positive at the 1% significance level, which indicates that institutional ownership may promote green innovation performance of the firm. Additionally, the coefficients of *Foreign sales* and *Industry competition* are insignificant.

#### 4.2.2. Moderating Effects Results

**Figure 2:**
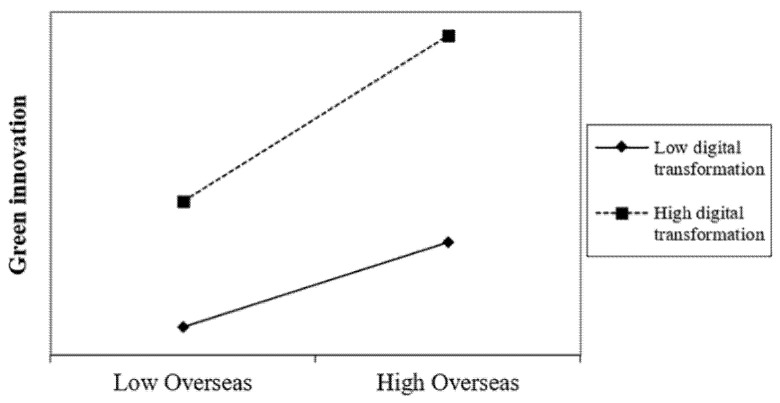
The moderating effect of digital transformation.

**Figure 3:**
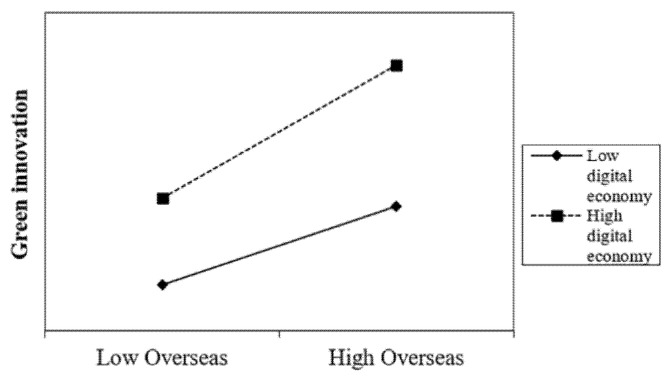
The moderating effect of digital economy.

As in Table 5, the results of moderating effects results are presented. As in Model 1, the coefficient of *Overseas* × *FD* is 0.146 and passes the 1% significance level test, which indicates that the degree of firm digitization also has a significant positive moderating effect on the main effect impact path. The higher the degree of firm digitization transformation, the greater the positive impact of the proportion of overseas experience of TMT on firm green innovation. Therefore, H2 is supported. In Model 2 of Table 5, the coefficient of *Overseas* × *RD* is 0.092 and passes the 1% significance level test, which indicates that the level of regional digital economy development positively moderates the relationship between TMTs’ overseas experience and green innovation performance. The higher the level of digital economy in the city where a firm is located, the greater the positive impact of TMTs’ overseas experience on firms’ green innovation; hence, H3 is supported. To better illustrate the moderating effects, we plot the relationship in Figure 2 and Figure 3, in which we can see that H2 and H3 are supported.

### 4.3. Robustness Checks

Firstly, this study uses an alternative measure to proxy for the key independent variable to test the robustness of our findings. We proxy the independent variable using a dummy variable of *Ifoverseas*, which takes the value of 1 if the TMT has any member with overseas experience and 0 otherwise. As indicated in Table 6, the robustness checks results are consistent with the main regression results. The main effects of the key independent variable and the interaction terms support our hypotheses, which indicates that our results are robust to the alternative measure of the independent variable.

Secondly, to alleviate the concern that our research design may face sample selection bias, that is, some firms may be more likely to hire TMTs with overseas experience and promote green innovation simultaneously, we conduct a two-stage Heckman selection model. In the Heckman model, the first step is a logit model to predict the possibility of the firm hiring overseas-experienced TMT and then to generate an inverse Mills ratio (*IMR*) to include in the second stage estimation. In the second step, we perform the baseline regressions with IMR included to check whether selection bias exists in the research and whether it exerts any impact on the main results. We include the same set of control variables as in the baseline model, as well as exclusive variables. Following previous works in the literature [52], we use the mean value of the proportion of TMTs’ overseas experience in the industry and the city as the exclusive variables because some industry or region characteristics may affect the firm’s decision to hire TMTs with overseas experience but may not affect green innovation outcome. Column 1 of Table 7 reports the first-stage estimation results. The coefficient of the exclusive variable is significantly positive, indicating the appropriateness of the variable. Column 2 of Table 7 reports the second-stage results, showing that the coefficient of *Overseas* remains significantly positive. These results indicate that our findings are robust after controlling for potential sample selection bias.

Finally, omitted variable bias or reverse causality may lead to endogeneity concerns in our study. Following previous studies [64,79], we employ the two-stage least square (2SLS) instrumental variable (IV) method to solve the endogeneity problem. We use the mean value of the proportion of TMTs’ overseas experience in the industry and the city as instrumental variables. These two variables are highly correlated with the *Overseas* and may not impact the dependent variable. All instruments pass the exogeneity and relevance tests. The results of the two stages of estimation are shown in Columns 3–4 of Table 7. The results of the first step are presented in Column 3 and the results of the second step are presented in Column 4. Our findings remain robust after we alleviate the potential concern over endogeneity.

## 5. Discussion

Nowadays, many countries have declared their commitment to sustainable practices in response to the United Nations’ SDGs. Meanwhile, the digital economy has fundamental impacts on business activities and performance. Especially in developing countries, such as China, Brazil and South Africa, firms are increasingly embedded in complex contexts that drive their strategic behavior and performance outcomes to be more environmentally friendly and sustainable [26,27]. As one of the key decision-makers in the firms, TMT plays a particularly important role in strategy formulation and the strategic direction process. TMTs’ overseas experience, as one type of unique resource, constitutes the cognitive basis of the team and thus influences firms’ strategic decision-making. Therefore, how TMT’s overseas experience affects firms’ green innovation in the digital era has become an important and interesting research direction.

By combining upper echelons theory with innovation research, this study sheds light on the theoretical relationship between TMT overseas experience, digital economy, and green innovation. To obtain an empirically grounded understanding of this important topic, this study uses a panel dataset of Chinese listed firms from 2011 to 2018, and finds significant and robust evidence to support the hypotheses. We have two important findings. First, TMT’s overseas experience positively affects firms’ green innovation performance in that the challenging and complex task of green innovation requires higher risk tolerance, confidence, and moral courage from decision makers, which are the at-tributes of TMT members with overseas experience. Second, firm- level digital transformation and region- level digital economy development positively moderate the relationship between TMT overseas experience and firms’ green innovation performance in that digital economy may reduce costs and improve efficiency.

### 5.1. Theoretical Implications

Synthesizing upper echelon theories and the emergent research on the digital economy, our study provides a theoretical framework and empirical evidence that TMTs’ overseas experience promotes firms’ green innovation performance and that this positive relationship is more pronounced for firms with higher digital transformation and for firms located in regions with better digital economy development. Thus, the contributions of this study are threefold.

Firstly, this study contributes to the existing literature on the antecedents and determinants of green innovation at the organizational level. Emergent research has focused on the determinants of green innovation/eco-innovation/environmental innovation from the perspectives of human capital [21], relationship system [36], export intensity [37], and dynamic capabilities [25]. We extend the literature on green innovation from the firm management perspective by investigating the interaction between TMT characteristics and digital economy development in the context of the digital age.

Secondly, this study advances the upper echelons literature on the impact of TMTs’ overseas experience on firms’ strategies and performance. Upper echelons theory is a theoretical framework that states that organizations’ strategic choices and performance are predicted by their TMT characteristics and a methodology that relies on TMT member demography as a measurement proxy for individual and group cognition and behavior [46,55]. Based on upper echelons theory, some previous studies have found the positive impacts of TMTs’ or CEOs’ international experience or diversity on firms’ strategic choices [43,48], internationalization [54,56], innovation performance [47,57], and social responsibility [58]. However, few studies have focused on TMTs’ international experience impacts on green performance, and most of the literature has not studied the role of digital economy development in the TMT’s decision-making process.

Finally, this study sheds light on the literature in a new field of research, digital economy and corporate strategy. Despite the growing awareness of the topic in practice, digital economy topics have rarely been touched upon in the previous literature on TMT. Previous studies have called for research to focus on the digital economy [11] and its impacts on innovation [12] and sustainable development [20]. To respond to this need, we contribute to determining the contingency effects of how firm digital transformation and regional digital economy shape the positive effect of TMTs’ overseas experience on green innovation.

### 5.2. Practical Implications

This study generates various policy implications for governments and managerial implications for firms that intend to promote sustainable growth and green transformation.

Firstly, our findings provide implications for governments, particularly in developing countries. Many countries have endeavored to fulfill the SDGs and should encourage more responsible innovation [2]. In developing countries, green innovation is of vital for firms to gain competitive advantages and be able to compete with developed countries [26]. This study reveals the importance of human capital in green innovation and extends research on green innovation in terms of the overseas experience of TMTs, which is helpful for firms when deciding on TMT strategies and investing in innovation. In this new stage of economic development, attracting excellent talent with overseas experience to drive green innovation is undoubtedly an important path toward achieving high-quality and environmentally friendly development. Additionally, governments should attach importance to digital economy development and further strengthen the development of digital infrastructure, which is particularly important for promoting firms to carry out energy savings, emission reduction, and environmentally friendly industrial upgrading.

Secondly, our findings suggest that the structure of the TMT is a key driver of green innovation performance. Since green innovation has been proven to be significant for firms’ development and performance [22,24], firms should consider its strategic aspects. Firms should be aware that heterogeneous TMTs may have richer perspectives and diversified resources and information to help make high-quality strategic decisions [51,80]. In addition, firms should be aware of the importance of green innovation in their organizations’ sustainable development.

Finally, this study highlights the value of digital economy for firms’ sustainable goals and sustainable development. It has been widely acknowledged that digital economy can aid in accelerating organizational performance [64,81] and sustainable development [14], but its role in the decision-making process of knowledge exchange and knowledge spillovers should not be neglected. Thus, firms should be able to recognize the importance of digital economy development in promoting green innovation and ensure that digital economy development plays a greater role in firms’ innovation and sustainable development. As firms are conducting green innovation in response to government policy, policy-makers should take preemptive action to encourage firms’ managers to pursue eco-innovation and green transformation in the digital era. To the extent that green innovation activities are advantageous for both firms and the whole society, this study implies that governments can promote firms’ green transformation and sustainable growth by providing financial incentives and environmental subsidies to achieve a win–win outcome.

### 5.3. Limitations and Future Research Directions

Although this study makes several contributions to the literature, there are some potential limitations that should be addressed in future research. Firstly, even though we conducted a thorough analysis using the latest measure of the digital economy (i.e., firms’ digital transformation by dictionary-based text analysis from annual report and regions’ digital economy development by PCA from statistical yearbooks), these measures can only reflect some specific aspects of digital economy development. Future research can extend our research with detailed studies of other specific components of digital transformation (e.g., digital marketing or digital servitization) or digital economy development (e.g., digital government or digital finance). Secondly, due to the limitation in data access, our research sample comprises Chinese listed firms, which are mostly large and profitable firms. Practice and previous studies show that there are potential differences between large firms and small- and medium-sized enterprises (SMEs) in terms of management practices and strategic decisions [82]. Future research can compare the different strategic outcomes between listed firms and SMEs in terms of TMT and green innovation using more in-depth survey data. Thirdly, our research has identified the TMT’s overseas background as a significant characteristic that shapes firms’ green innovation. Limited by data access, the key measure of TMT’s overseas background is based on the general calculation on the quantity of TMT background based on the text analysis from the annual report. If future research conducts survey analysis and obtain more detailed data, it would be interesting to examine more diverse aspects of TMT members regarding overseas background (e.g., local connections and inter-organization connections) to compare with local TMT members. Moreover, it would be interesting to investigate the original countries of the TMTs’ overseas experience because the context in which that overseas experience is absorbed may matter in the green innovation decision-making process. Last but not least, as it has been documented in the literature that green innovation requires more external sources of knowledge and information compared to other innovations [83], exploring the differential impacts of internal and external sources of knowledge on green innovation deserves further investigation. Examining the organizational absorptive capacity of external knowledge (e.g., partnerships, cooperation, and open innovation) is worthy of further study.

## 6. Conclusions

Combining the upper echelon theory and innovation research, this study formulates a theoretical framework to analyze the impact of TMTs’ overseas experience on firms’ green innovation performance and the moderating role of digital economy. By using a panel dataset of Chinese listed firms covering 2011 to 2018, this study finds significant and robust empirical evidence to support the hypotheses. It shows that TMTs with more overseas experience is indeed helpful for firms to develop green innovation technologies. We further find that firm digital transformation and region digital economy development positively moderate the relationship between TMTs’ overseas experience and firms’ green innovation performance. Our findings extend the research related to TMT and green innovation by investigating the interactive roles of TMT overseas experience and digital economy development. We also provide important implications for governments and managers to encourage sustainable development.

## Figures and Tables

**Table 1:** Definitions of variables.

	Symbols	Definitions
Dependent variable	*GI*	The logarithm of the total number of green patents applied of the firm
Independent variable	*Oversea* *s*	The proportion of TMT members with overseas experience to the total number of TMT members
Moderators	*FD*	The logarithm of the total word frequency of terms including blockchain technology, big data technology, artificial intelligence technology and cloud computing technology appearing in the annual report
	*RD*	Region-level digital economy development index
Control variables	*Firm* *size*	The logarithm of a firm’s total assets
	*Firm age*	The logarithm of the firm’s age
	*R* *OA*	The return to total assets
	*R&D intensity*	The share of annual R&D investment to annual operating revenue
	*Foreign sales*	The ratio of overseas income to annual total income
	*CEO* *duality*	A dummy variable with a value of 1 if the CEO and chairman is the same person and 0 otherwise
	*Fund ownership*	The share of ownership owned by funds
	*Industry competition*	One minus the HHI index of the industry

**Table 2:** Descriptive statistics.

Variable	Observations	Mean	Standard Deviation
*GI*	16,246	0.283	0.658
*Overseas*	16,246	0.047	0.104
*FD*	16,246	0.862	1.200
*RD*	16,246	1.242	1.736
*Firm size*	16,246	22.048	1.265
*Firm age*	16,246	2.787	0.376
*ROA*	16,246	0.040	0.217
*R&D intensity*	16,246	0.034	0.097
*Foreign sales*	16,246	0.122	0.196
*CEO duality*	16,246	0.271	0.444
*Fund ownership*	16,246	1.055	0.928
*Industry competition*	16,246	0.732	0.085

**Table 3:** Correlation Matrix.

Variable	*GI*	*Overseas*	*FD*	*RD*	*Firm Size*	*Firm Age*	*ROA*	*R&D Intensity*	*Foreign Sales*	*CEO Duality*	*Fund Ownership*	*Industry Competition*
*GI*	1.000											
*Overseas*	0.059 ***	1.000										
*FD*	0.136 ***	0.110 ***	1.000									
*RD*	0.090 ***	0.141 ***	0.262 ***	1.000								
*Firm size*	0.221 ***	0.053 ***	0.055 ***	0.029 ***	1.000							
*Firm age*	−0.050 ***	−0.054 ***	−0.030 ***	0.017 **	0.156 ***	1.000						
*ROA*	0.014 **	0.000	0.005	0.021 ***	−0.007	−0.025 ***	1.000					
*R&D intensity*	0.082 ***	0.074 ***	0.168 ***	0.086 ***	−0.125 ***	−0.087 ***	−0.011	1.000				
*Foreign sales*	0.039 ***	0.154 ***	−0.020 ***	0.062 ***	−0.078 ***	−0.074 ***	−0.006	0.047 ***	1.000			
*CEO duality*	0.004	0.067 ***	0.083 ***	0.096 ***	−0.187 ***	−0.116 ***	0.024 ***	0.087 ***	0.088 ***	1.000		
*Fund ownership*	0.113 ***	0.062 ***	0.115 ***	0.008	0.223 ***	−0.034 ***	0.074 ***	0.042 ***	0.000	−0.011	1.000	
*Industry competition*	0.005	−0.021 ***	0.026 ***	0.021 ***	−0.040 ***	−0.004	0.013 *	0.028 ***	0.053 ***	0.020 ***	0.033 ***	1.000

Note: * *p* < 0.10, ** *p* < 0.05, *** *p* < 0.01.

**Table 4:** Baseline Regression Results.

Variables	(1)	(2)	(3)
*Overseas*		0.128 **	0.109 *
		(0.058)	(0.060)
*FD*			0.078 ***
			(0.005)
*RD*			0.013 ***
			(0.004)
*Firm size*	0.165 ***	0.164 ***	0.125 ***
	(0.005)	(0.005)	(0.005)
*Firm age*	−0.039 **	−0.038 **	−0.119 ***
	(0.016)	(0.016)	(0.017)
*ROA*	0.054 **	0.054 **	0.042 *
	(0.024)	(0.024)	(0.025)
*R&D intensity*	0.219 ***	0.215 ***	0.605 ***
	(0.070)	(0.070)	(0.072)
*Foreign sales*	0.031	0.041	0.055
	(0.032)	(0.032)	(0.031)
*CEO duality*	0.025 *	0.024 *	0.028 **
	(0.013)	(0.013)	(0.014)
*Fund ownership*	0.058 ***	0.058 ***	0.064 ***
	(0.007)	(0.007)	(0.007)
*Industry competition*	0.083	0.083	0.226
	(0.122)	(0.122)	(0.168)
Constant	−3.346 ***	−3.344 ***	−2.488 ***
	(0.152)	(0.152)	(0.127)
*N*	13,560	13,560	13,560
*adj. R* ^2^	0.187	0.188	0.198

Note: a. Robust standard errors in parentheses; b. * *p* < 0.10, ** *p* < 0.05, *** *p* < 0.01.

**Table 5:** Moderating model estimation.

Variables	(1)	(2)
*Overseas*	0.064	0.072
	(0.074)	(0.075)
*FD*	0.047 ***	0.055 ***
	(0.007)	(0.006)
*RD*	0.013 ***	0.007 *
	(0.004)	(0.004)
*Overseas × FD*	0.146 ***	
	(0.043)	
*Overseas × RD*		0.092 ***
		(0.027)
*Firm size*	0.158 ***	0.158 ***
	(0.005)	(0.005)
*Firm age*	−0.026	−0.027 *
	(0.016)	(0.016)
*ROA*	0.050 **	0.049 **
	(0.024)	(0.024)
*R&D intensity*	0.206 ***	0.205 ***
	(0.070)	(0.070)
*Foreign sales*	0.028	0.025
	(0.032)	(0.032)
*CEO duality*	0.016	0.014
	(0.013)	(0.013)
*Fund ownership*	0.056 ***	0.056 ***
	(0.007)	(0.007)
*Industry competition*	0.068	0.078
	(0.122)	(0.122)
Constant	−3.280 ***	−3.283 ***
	(0.152)	(0.152)
*N*	13,560	13,560
*adj. R* ^2^	0.194	0.194

Note: a. Robust standard errors in parentheses; b. * *p* < 0.10, ** *p* < 0.05, *** *p* < 0.01.

**Table 6:** Robustness Checks: Alternative Proxy for TMTs’ Overseas Experience.

Variable	(1)	(2)	(3)	(4)	(5)
*Ifoverseas*		0.057 ***	0.047 ***	0.006	0.016
		(0.014)	(0.014)	(0.018)	(0.019)
*FD*			0.055 ***	0.047 ***	0.054 ***
			(0.006)	(0.007)	(0.006)
*RD*			0.012 ***	0.012 ***	0.002
			(0.004)	(0.004)	(0.004)
*Ifoverseas × FD*				0.030 ***	
				(0.011)	
*Ifoverseas × RD*					0.037 ***
					(0.007)
*Firm size*	0.165 ***	0.163 ***	0.157 ***	0.157 ***	0.157 ***
	(0.005)	(0.005)	(0.005)	(0.005)	(0.005)
*Firm age*	−0.039 **	−0.036 **	−0.024	−0.024	−0.026
	(0.016)	(0.016)	(0.016)	(0.016)	(0.016)
*ROA*	0.054 **	0.054 **	0.049 **	0.051 **	0.049 **
	(0.024)	(0.024)	(0.024)	(0.024)	(0.024)
*R&D intensity*	0.219 ***	0.212 ***	0.203 ***	0.200 ***	0.203 ***
	(0.070)	(0.070)	(0.070)	(0.070)	(0.070)
*Foreign sales*	0.031	0.047	0.034	0.032	0.032
	(0.032)	(0.032)	(0.032)	(0.032)	(0.032)
*CEO duality*	0.025 *	0.022 *	0.014	0.013	0.013
	(0.013)	(0.013)	(0.013)	(0.013)	(0.013)
*Fund ownership*	0.058 ***	0.057 ***	0.055 ***	0.055 ***	0.056 ***
	(0.007)	(0.007)	(0.007)	(0.007)	(0.007)
*Industry competition*	0.083	0.085	0.072	0.069	0.075
	(0.122)	(0.122)	(0.122)	(0.122)	(0.122)
cons	−3.346 ***	−3.329 ***	−3.272 ***	−3.257 ***	−3.263 ***
	(0.152)	(0.152)	(0.152)	(0.152)	(0.152)
*N*	13,565	13,565	13,565	13,565	13,565
*adj. R* ^2^	0.187	0.188	0.194	0.195	0.194

Note: a. Robust standard errors in parentheses; b. * *p* < 0.10, ** *p* < 0.05, *** *p* < 0.01.

**Table 7:** Robustness checks: 2-stage Heckman model and 2SLS.

	(1)	(2)	(3)	(4)
	Heckman Model		2SLS	
Variables	*Ifoverseas*	*GI*	*Overseas*	*GI*
*Overseas*		0.117 **		1.390 ***
		(0.060)		(0.187)
*Firm size*	0.194 ***	0.035 ***	0.003 ***	0.125 ***
	(0.039)	(0.009)	(0.001)	(0.005)
*Firm age*	−0.785 ***	0.131 **	−0.008 ***	−0.126 ***
	(0.134)	(0.061)	(0.002)	(0.017)
*ROA*	−0.177	−0.000	−0.004	0.049 *
	(0.395)	(0.016)	(0.003)	(0.026)
*R&D intensity*	−0.007	0.052	0.026 ***	0.676 ***
	(0.199)	(0.048)	(0.010)	(0.074)
*Foreign sales*	0.719 ***	0.051	0.067 ***	0.034
	(0.209)	(0.042)	(0.004)	(0.035)
*CEO duality*	0.084	0.007	0.008 ***	0.028 **
	(0.077)	(0.014)	(0.002)	(0.014)
*Fund ownership*	−0.004	−0.010 *	0.004 ***	0.066 ***
	(0.033)	(0.005)	(0.001)	(0.007)
*Industry competition*	0.068	0.106	0.013	0.320 ***
	(0.394)	(0.075)	(0.017)	(0.070)
*Mean_Overseas_ind*	18.800 ***		0.776 ***	
	(1.720)		(0.072)	
*Mean_Overseas_city*	31.964 ***		0.910 ***	
	(1.410)		(0.026)	
*IMR*		0.010		
		(0.007)		
Constant	−7.346 ***	−0.954 ***	−0.074 ***	
	(0.832)	(0.260)	(0.018)	
*N*	13,560	13,560	13,560	13,560

Note: a. Standard errors in parentheses; b. * *p* < 0.10, ** *p* < 0.05, *** *p* < 0.01.

## Data Availability

Data available in the chargeable databases China Security Market and Accounting Research (CSMAR) database.
